# Ulvan Polysaccharide of *Ulva lactuca* From Coastal Indonesia: Functional Prebiotic Properties via Enzymatic and Nonenzymatic Extraction

**DOI:** 10.1155/ijfo/4529168

**Published:** 2026-04-30

**Authors:** Yuliana Tandi Rubak, Herianus J. D. Lalel, Budiana I. Gusti M. Ngurah, Welem Linggi Turupadang, Fenny Amilia Mahara, Dandy Yusuf

**Affiliations:** ^1^ Agrotechnology Study Program, Faculty of Agriculture, Universitas Nusa Cendana, Kupang, Indonesia, undana.ac.id; ^2^ Chemistry Study Program, Faculty of Education and Teacher Training, Universitas Nusa Cendana, Kupang, Indonesia, undana.ac.id; ^3^ Aquaculture Study Program, Faculty of Animal Husbandry, Marine and Fisheries, Universitas Nusa Cendana, Kupang, Indonesia, undana.ac.id; ^4^ Research Center for Food Technology and Processing, National Research and Innovation Agency (BRIN), Yogyakarta, Indonesia, brin.go.id; ^5^ Research Center for Applied Microbiology, National Research and Innovation Agency (BRIN), Cibinong, Indonesia, brin.go.id; ^6^ Research Collaboration Center for Traditional Fermentation, Surakarta, Indonesia

**Keywords:** antioxidant properties, enzyme-assisted extraction, lactic acid bacteria fermentation, prebiotic potential, *Ulva lactuca*, ulvan polysaccharide

## Abstract

Indonesia′s rich macroalgal biodiversity offers valuable resources for functional food development, particularly ulvan, a sulfated polysaccharide derived from *Ulva lactuca*. While ulvan shows promising prebiotic potential, its extraction efficiency and structure–function relationships are strongly influenced by processing conditions. This study optimized ulvan recovery from *U. lactuca* harvested from the Bolok coastal waters (Kupang, Indonesia) and evaluated its chemical composition, structural characteristics, and in vitro prebiotic functionality. Three extraction strategies were compared: mild hydrothermal–enzymatic (cellulase)–ultrasonication (HEU‐C), mild hydrothermal–multienzyme–ultrasonication (HEU‐Mix), and a nonenzymatic hydrothermal–ultrasonication (HU) method. The HEU‐C method achieved the highest yield (29.85%), significantly exceeding that of HU (12.22%). Chemical analyses revealed that HEU‐C extracts contained the highest levels of polysaccharides, reducing sugars, and uronic acids, whereas HU extracts retained the greatest sulfate content. FTIR and SEM analyses demonstrated that different extraction methods induced distinct structural modifications and changes in surface morphology. Ulvan extracted via HEU‐C exhibited the strongest antioxidant activity (DPPH radical scavenging capacity: 50.58%), followed by HEU‐Mix and HU. In vitro fermentation assays showed that HEU‐C extracts (1%–5% *w*/*v*) significantly promoted the growth of *Lactobacillus rhamnosus* RAL43, *Lactobacillus plantarum* RAL25, and *Lactococcus lactis* SwR14, with performance comparable to or exceeding that of fructooligosaccharides. Fermentation was further characterized by soluble sugar consumption and the production of short‐chain fatty acids (SCFAs), including acetic, propionic, and butyric acids. Overall, enzyme‐assisted extraction enhanced ulvan bioactivity, structural accessibility, and prebiotic performance, positioning it as a promising functional ingredient for microbiota‐targeted food applications and marine‐derived nutraceutical development.

## 1. Introduction

Indonesia′s extensive coastline supports one of the world′s richest macroalgal biodiversity profiles, with more than 325 species documented across 24 provinces, establishing the country as the second‐largest seaweed producer globally [1]. This ecological and economic potential underscores macroalgae as strategic biological resources for the development of high‐value bioproducts, including functional food ingredients, pharmaceuticals, and cosmeceuticals [2–6].

Among dominant green macroalgae, *Ulva lactuca*, commonly known as sea lettuce, is widely distributed and characterized by high levels of carbohydrates, proteins, minerals, vitamins, and diverse phytochemicals such as flavonoids, tannins, terpenoids, and phenolics [7–9]. These bioactive constituents contribute to a wide range of biological activities, including antioxidant, antimicrobial, and anti‐inflammatory effects [5, 10–12]. Structurally, *Ulva* cell walls consist of cellulose, xyloglucan, glucuronan, and ulvan, with ulvan accounting for approximately 13%–39% of the dry biomass [11, 13–15].

Ulvan is a sulfated, polyanionic heteropolysaccharide composed primarily of rhamnose, glucuronic acid, iduronic acid, and xylose, organized as repeating disaccharide units linked through *α*‐ and *β*‐(1⟶4) glycosidic bonds [16–18]. Its hydrophilic and semicrystalline structure resists digestion in the upper gastrointestinal tract, enabling it to reach the colon and function as a selective substrate for beneficial gut microorganisms [19, 20]. Consequently, ulvan has gained attention as a promising natural prebiotic, supported by in vitro and in vivo studies demonstrating enhanced growth of probiotic *Lactobacillus* and *Bifidobacterium*, increased short‐chain fatty acid (SCFA) production, improved gut microbial balance, and immune modulation [18, 21–26].

The prebiotic efficacy of ulvan is strongly influenced by its structural attributes, monosaccharide composition, degree of sulfation, molecular weight, and backbone conformation, parameters that are highly dependent on the extraction process [4, 16, 18, 27–29]. Various extraction strategies, including hydrothermal, chemical, ultrasound‐assisted, microwave‐assisted, and enzyme‐assisted methods, have been employed to disrupt *Ulva* cell walls and recover ulvan [13, 29–37]. However, harsh physical or chemical conditions often induce desulfation and depolymerization, thereby compromising ulvan structural integrity and functionality [18]. In contrast, enzyme‐assisted extraction (EAE) has gained increasing attention due to its selectivity, higher purity yields, and ability to preserve structural features while generating low‐ to medium‐molecular‐weight ulvan fractions that are more readily fermentable by probiotic bacteria [16, 29, 38].

Despite growing interest in ulvan‐based prebiotics, comparative studies on enzymatic and nonenzymatic extraction of *U. lactuca* from Indonesia, one of the world′s most productive seaweed regions, remain limited. Moreover, the relationship between extraction‐induced structural variations and subsequent prebiotic functionality has not been sufficiently elucidated. Therefore, this study is aimed at characterizing ulvan‐rich polysaccharide extracts from *U. lactuca* harvested from coastal Indonesia using enzymatic (cellulase and a cellulase–pectinase–protease mixture) and nonenzymatic mild hydrothermal–ultrasonic extraction methods. Furthermore, the prebiotic potential of these extracts was evaluated using three candidate probiotic lactic acid bacteria (LAB): *Lactobacillus rhamnosus* RAL43, *Lactobacillus plantarum* RAL25, and *Lactococcus lactis* SwR14, previously isolated and characterized in our earlier studies [39–41].

## 2. Materials and Methods

### 2.1. Preparation of *U. lactuca* Biomass

Fresh, bright green thalli of *U. lactuca* were collected from the Bolok coastal waters of Kupang, East Nusa Tenggara, Indonesia, under typical marine salinity conditions (32‰–34‰) and warm tropical temperatures (28°C–30°C). The samples were thoroughly rinsed with clean water to remove debris and residual seawater. The biomass was shade‐dried for 3–4 days, cut into 2–3‐cm pieces, ground, and sieved through a 60‐mesh screen to obtain a powder. The resulting powder was stored in sealed polyethylene bags at room temperature in the dark until extraction.

### 2.2. Extraction of Ulvan‐Rich Polysaccharides

Ulvan‐rich polysaccharides were extracted from *U. lactuca* biomass powder using enzymatic and multienzyme‐assisted approaches based on the methods described by Wang et al. [38] and Romero Malvis et al. [29], with slight modifications. Three extraction methods were applied: (1) mild hydrothermal–enzymatic (cellulase)–ultrasonication (HEU‐C), (2) mild hydrothermal–enzymatic (cellulase–pectinase–protease)–ultrasonication (HEU‐Mix), and (3) mild hydrothermal–ultrasonication without enzymes (HU).

Dried *U. lactuca* powder (20 g) was suspended in deionized water at a ratio of 1:20 (*w*/*v*) and heated at 50°C for 30 min to soften the cell wall matrix. For the HEU‐C method, the suspension pH was adjusted to 5.0 using 0.2 M acetate buffer, followed by the addition of cellulase (12 mL, 1.2 U/mL; Sigma‐Aldrich, United States). The mixture was incubated at 50°C and 150 rpm for 4 h in a shaking incubator (BioShaker TAITEC BR‐43FH, Tokyo, Japan).

For the HEU‐Mix treatment, after 3 h of cellulase incubation, the pH was adjusted to 7.0, followed by the addition of pectinase (1 mg/g biomass) and protease (0.5 mg/g biomass), and incubation was continued for an additional 1 h. The HU method was performed without enzyme addition or incubation.

All suspensions were subsequently subjected to ultrasonication using an ultrasonic bath (Ultrasonic Homogenizer UH 150, SMT Co. Ltd.) at 70°C for 30 min. The mixtures were filtered through cotton gauze and centrifuged at 8000 × *g* for 10 min at 4°C. The resulting supernatants were precipitated by adding four volumes of cold 96% ethanol and stored at 4°C for 24 h. The precipitates were recovered by centrifugation (8000 × *g*, 10 min, 4°C), washed twice with 96% ethanol, dried at 50°C, and sieved through an 80‐mesh sieve.

Extraction yield was calculated as follows:

Yield %=dry polysaccharide weightdry biomass weight×100.

All extractions were performed in triplicate. The dried ulvan extracts were stored in airtight containers at room temperature until further analysis.

### 2.3. Chemical Characterization of Ulvan Extract

Major chemical components of the ulvan extract were quantified using established colorimetric assays. Total polysaccharides were determined using the phenol–sulfuric acid method [42] at 490 nm, with glucose as the calibration standard. Reducing sugars were measured using the dinitrosalicylic (DNS) acid assay [43] at 540 nm. Uronic acid content was quantified using the carbazole colorimetric method [44] at 530 nm, with galacturonic acid as the standard. Sulfate content was determined by the BaCl_2_–gelatin turbidimetric method [45], using sodium sulfate as the reference compound. Antioxidant activity was evaluated using the DPPH radical scavenging assay [46] at 517 nm.

### 2.4. Structural and Morphological Characterization of the Ulvan Extract

#### 2.4.1. Scanning Electron Microscopy (SEM)

Surface morphology of ulvan extracts obtained from the three extraction methods was examined using a benchtop SEM (JSM‐6510LA, JEOL, South Korea). Samples were sputter‐coated with gold at 20 mA for 60 s. SEM observations were performed at an accelerating voltage of 3 kV, 30% signal intensity, and an aperture size of 4 under high‐vacuum conditions using secondary electron detection. Images were acquired at magnifications of 1000×, 5000×, and 10,000×.

#### 2.4.2. Fourier Transform Infrared (FTIR) Spectroscopy

Ulvan extracts obtained using nonenzymatic (HU) and enzymatic extraction methods were analyzed by FTIR spectroscopy to assess functional group composition. Spectra were recorded using a TENSOR II FTIR spectrophotometer (Bruker Optics, United States) over the range of 4000–400 cm^−1^ at a resolution of 4 cm^−1^.

### 2.5. In Vitro Evaluation of the Prebiotic Potential of *U. lactuca* Polysaccharides (ULPs)

#### 2.5.1. Rejuvenation and Confirmation of LAB Isolates

The probiotic candidate LAB strains used in this study were *Lb. rhamnosus* RAL43 and *Lb. plantarum* RAL25, isolated from Indonesian kefir grains [40, 41], and *Lc. lactis* SwR14, isolated from the fermented pork product Su′i Wuu [39]. These strains were evaluated both individually and as a mixed culture (RAL43+RAL25+SwR14) to assess the prebiotic potential of ULPs. Prior to experimentation, each isolate was rejuvenated through two successive transfers in de Man, Rogosa, and Sharpe broth (MRSB) (GM369, HiMedia Laboratories, Mumbai, India) and incubated at 37°C for 24 h. Culture identity and purity were confirmed based on colony morphology, Gram staining, and catalase testing.

#### 2.5.2. Preparation of Glucose‐Free Modified MRSB Supplemented With ULP

A glucose‐free modified MRSB was prepared according to standard formulations, with glucose omitted to eliminate alternative carbon sources. The medium was adjusted to pH 7.0 and sterilized at 121°C for 15 min. ULPs obtained via HEU‐C and HEU‐Mix methods were dissolved in sterile distilled water to final concentrations of 1.0%, 2.5%, and 5.0% (*w*/*v*), sterilized by filtration through a 0.22‐*μ*m nylon membrane filter, and aseptically added to the cooled medium. Fructooligosaccharides (FOSs) (2.5% *w*/*v*; Sigma‐Aldrich, United States) were prepared similarly and used as the reference prebiotic control. A basal glucose‐free modified MRSB without any added carbohydrate or carbon source was used as a negative control.

#### 2.5.3. In Vitro Fermentation Assay

In vitro fermentation was conducted to assess LAB growth, soluble sugar utilization, and SCFA production. Each fermentation tube containing 10 mL of supplemented glucose‐free MRSB was inoculated with 1 mL of actively growing LAB culture (~9% *v*/*v*). Starter cultures were propagated in MRSB for 24 h, reaching cell densities of approximately 8.0–9.5 log CFU/mL, resulting in an estimated initial inoculum level of ~7.0–8.5 log CFU/mL at 0 h.

For mixed‐culture fermentations, equal volumes of each LAB strain were combined prior to inoculation to obtain a composite starter culture. Cultures were incubated at 37°C for 24 h. Bacterial viability was determined at 0 and 24 h by the plate counting method on MRS agar and expressed as log colony‐forming units per milliliter.

Soluble sugar content was determined by measuring total sugars and reducing sugars in the fermentation supernatant using the phenol–sulfuric acid and DNS methods, respectively. SCFA analysis was performed according to Calvigioni et al. [47] with minor modifications. Briefly, fermentation broth was centrifuged at 8500 rpm for 10 min at 4°C, and the supernatant was filtered through a 0.22‐*μ*m nylon membrane filter. Filtrates were transferred into autosampler vials, and 20 *μ*L aliquots were injected into a Shimadzu HPLC system equipped with a C18 column (150 × 4.6 mm, 3–5 *μ*m; Shimadzu Shim‐Pack VP‐ODS) and a refractive index detector (RID)‐20A.

The mobile phase, consisting of water with 0.1% phosphoric acid and acetonitrile with 0.1% formic acid, was delivered at a flow rate of 0.5 mL/min. Identification of acetic, propionic, and butyric acids was based on their respective retention times compared to external standards (Sigma‐Aldrich, United States). Quantification was performed using external standard curves at concentrations of 10, 100, and 1000 ppm.

### 2.6. Data Analysis

All data are presented as mean ± standard deviation from three independent experiments. Statistical analyses were performed using SPSS Version 20.0 (IBM Corp., Armonk, New York, United States), with significance set at *p* < 0.05. Differences among treatments were evaluated using one‐way ANOVA followed by Tukey′s HSD post hoc test. Pairwise comparisons between HEU‐C and HEU‐Mix treatments were conducted using independent‐samples *t*‐tests.

## 3. Results and Discussion

### 3.1. Extraction Yield

To compare the efficiency of the extraction strategies, the total yield was quantified across the three treatments (Table 1). EAEs produced significantly higher yields (29.85*%* ± 3.29*%* for HEU‐C and 27.50*%* ± 3.28*%* for HEU‐Mix) than the nonenzymatic hydrothermal–ultrasonication method (12.22*%* ± 0.33*%* for HU), demonstrating that enzymatic depolymerization substantially enhances ulvan recovery. These values fall within the reported range for sulfated seaweed polysaccharides (8%–29% of dry weight), which varies depending on extraction conditions Li et al. [48]. No significant difference was observed between the single cellulase treatment (HEU‐C) and the mixed‐enzyme system (HEU‐Mix).

**Table 1:** Total yield and chemical composition of ulvan extract.

Treatment	Total yield (%)	Total polysaccharides (mg/mL)	Reducing sugar (mg/mL)	Uronic acid (*μ*g/mg)	Sulfate (%)
HEU‐C	29.85 ± 3.29^b^	13.83 ± 0.58^b^	3.01 ± 0.04^c^	39.56 ± 10.01^b^	1.97 ± 1.11^a^
HEU‐Mix	27.50 ± 3.28^b^	9.41 ± 0.52^a^	1.28 ± 0.05^a^	22.11 ± 1.92^a^	4.34 ± 0.48^ab^
HU	12.22 ± 0.33^a^	8.26 ± 0.17^a^	1.84 ± 0.01^b^	33.22 ± 5.09^ab^	6.57 ± 1.11^b^

*Note:* Different superscript letters (a–c) within the same column indicate statistically significant differences between treatments (*p* < 0.05).

Abbreviations: HEU‐C, mild hydrothermal–enzymatic (cellulase)–ultrasonication; HEU‐Mix, mild hydrothermal–enzymatic (cellulase–pectinase–protease)–ultrasonication; HU, mild hydrothermal–ultrasonication without enzymes.

The superior performance of HEU‐C highlights the effectiveness of cellulase, a *β*‐glucosidase targeting *β*‐(1⟶4) and *β*‐(1⟶3) linkages, in weakening the cellulose–hemicellulose matrix of *U. lactuca*, thereby improving access to cell wall–bound ulvan. This mechanism aligns with the findings of Romero Malvis et al. [29] and Kidgell et al. [49]. Furthermore, the extraction yield obtained in this study is comparable to or higher than previously reported cellulase‐assisted ultrasonic extractions, such as 26.7*%* ± 0.9*%* in Chen et al. [16] and 14.46% in Wang et al. [38].

Despite its broader catalytic spectrum, the multienzyme system (HEU‐Mix) did not outperform cellulase alone (HEU‐C). Although such systems may theoretically exhibit synergistic degradation of complex matrices [18, 29], factors such as enzyme competition, substrate preference, or overhydrolysis may restrict their overall efficiency [38]. Excessive depolymerization may also generate smaller ulvan fragments that reduce the recoverable final mass [18, 49]. The yield observed for HEU‐Mix is consistent with ultrasound‐assisted multienzyme extractions previously reported for *U. lactuca* (30.14% [38]). Beyond enzyme specificity, biomass variability, thermal profile, and solvent interactions also influence extraction efficiency [27].

In contrast, HU produced the lowest yield, reflecting its reliance solely on physical disruption. Although hydrothermal softening and ultrasonic cavitation can increase tissue permeability and solvent penetration, they are insufficient to fully liberate ulvan from protein–pectin complexes without enzymatic assistance. Consequently, ulvan recovery remained limited, in agreement with earlier nonenzymatic hydrothermal or ultrasonic extractions that reported yields of only 10%–22% [13, 31, 49].

### 3.2. Chemical Composition

Chemical composition analysis revealed distinct differences among the extraction treatments (Table 1). HEU‐C extracts contained significantly higher levels of total polysaccharides (13.83 ± 0.58 mg/mL), reducing sugars (3.01 ± 0.04 mg/mL), and uronic acids (39.56 ± 10.01 * μ*g/mg) than both HEU‐Mix and HU. This pattern indicates that cellulase‐assisted disruption effectively released glucans and uronic acid–rich polysaccharides embedded within the *Ulva* cell wall matrix. In contrast, HU showed the highest sulfate content (6.57*%* ± 1.11*%*), whereas both enzymatic treatments exhibited significantly lower sulfate levels, likely reflecting partial desulfation or the preferential solubilization of less‐sulfated fractions during enzymatic processing.

The high carbohydrate content observed in HEU‐C aligns with previous reports demonstrating that cellulase enhances the release of polysaccharide‐ and ulvan‐associated sugars during extraction [21, 38]. Reducing sugar levels followed a similar trend, with HEU‐C producing the highest concentration, consistent with cellulase‐mediated cleavage of *β*‐(1⟶4) linkages that generate additional reducing ends [38, 49]. In contrast, HEU‐Mix yielded lower reducing sugar levels, possibly due to the formation of higher molecular weight complexes or the partial reassociation of polysaccharide fragments despite the broader enzyme spectrum. HU generated intermediate reducing sugar levels, consistent with nonspecific chain scission induced by hydrothermal softening and ultrasonic cavitation.

Uronic acid, a key contributor to ulvan′s anionic character and bioactivity, also varied significantly across treatments. HEU‐C yielded the highest uronic acid concentration, suggesting enhanced release of uronate‐rich domains following cellulase‐mediated loosening of cellulose microfibrils. HEU‐Mix showed significantly lower uronic acid content, which may reflect excessive hydrolysis of pectin–protein complexes or the fragmentation of uronate‐containing regions. HU produced substantial uronic acid levels, facilitated by hydrothermal and ultrasonic processes that sufficiently disrupt cell wall structures to solubilize these components.

Sulfate content exhibited the opposite pattern (HU > HEU‐Mix > HEU‐C). Sulfate ester groups are known to be sensitive to enzymatic cleavage; furthermore, desulfated or low‐molecular‐weight sulfated fragments may remain in the supernatant during ethanol precipitation, thereby reducing the measurable sulfate content in enzymatic extracts [29, 49]. Nonenzymatic extraction, by contrast, tends to better preserve native sulfation patterns. Reported sulfate levels in ulvan vary widely (2.350%) depending on species, seasonal variation, and extraction conditions [28, 36, 50]. Taken together, these compositional differences suggest that enzymatic treatments produce selectively degraded ulvan enriched in glucan‐ and uronate‐containing fractions, whereas physical extraction more effectively preserves highly sulfated components.

### 3.3. Antioxidant Activity of Ulvan Extracts

**Figure 1:**
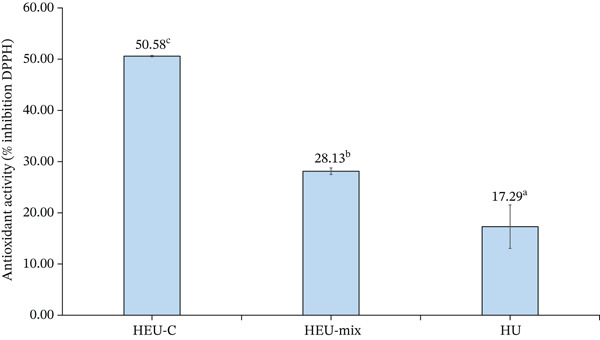
DPPH radical scavenging activity of ULPs (*Ulva lactuca* polysaccharides) obtained using different extraction methods. HEU‐C, mild hydrothermal–enzymatic (cellulase)–ultrasonication; HEU‐Mix, mild hydrothermal–enzymatic (cellulase–pectinase–protease)–ultrasonication; HU, mild hydrothermal–ultrasonication without enzymes. Different superscript letters (a–c) above the bars indicate statistically significant differences between treatments (*p* < 0.05).

The antioxidant activity of ulvan extracts varied significantly depending on the extraction method applied (Figure 1). Ulvan obtained via HEU‐C exhibited the highest DPPH radical scavenging activity (50.58*%* ± 0.14*%*). In contrast, HEU‐Mix extracts showed lower activity (28.13*%* ± 0.65*%*), which may be attributed to the partial degradation of polysaccharide chains or enzyme–enzyme interactions that reduced the antioxidant efficacy. HU extracts, obtained without enzymatic assistance, displayed the lowest scavenging activity (17.29*%* ± 4.26*%*), indicating that the absence of enzymatic treatment limits the release of antioxidant‐active compounds from *U. lactuca*. These results are consistent with previous studies reporting enhanced antioxidant activity (up to 69.80%) when ultrasonic and enzymatic extraction methods are combined [29, 38].

Ulvan exerts antioxidant effects primarily through hydrogen or electron donation to neutralize free radicals and through chelation of transition metal ions that catalyze Fenton or Haber–Weiss reactions [51, 52]. Antioxidant capacity is further influenced by the presence of associated phenolics, polyphenols, and flavonoids, which enhance radical scavenging activity [53–55]. In addition, amino acids and proteins present in *U. lactuca* may contribute to antioxidant activity via metal chelation and radical scavenging mechanisms [29]. Structural features of ulvan, particularly sulfate and uronate groups, have also been reported to enhance antioxidant reactivity [51]. The synergy between enzymatic hydrolysis and ultrasonication likely facilitates improved accessibility and release of these bioactive compounds, thereby enhancing antioxidant performance [38, 56]. Finally, a concentration‐dependent effect was observed, with higher extract concentrations corresponding to increased radical scavenging activity [38, 57].

### 3.4. SEM Analysis

**Figure 2:**
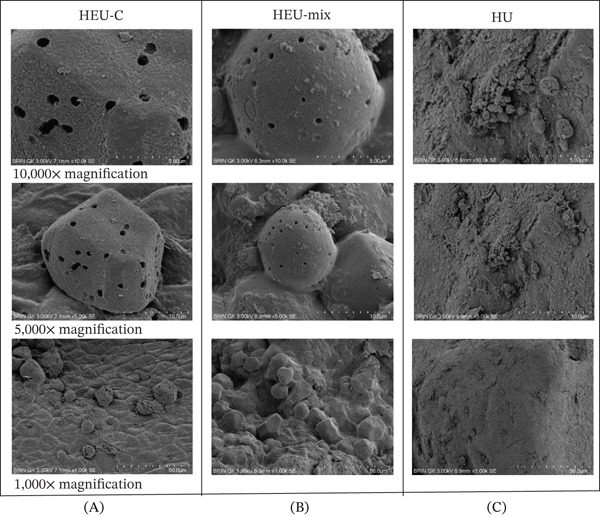
SEM micrographs of ULPs (*Ulva lactuca* polysaccharides) showing surface morphology differences among extraction treatments: (A) HEU‐C, (B) HEU‐Mix, and (C) HU. HEU‐C, mild hydrothermal–enzymatic (cellulase)–ultrasonication; HEU‐Mix, mild hydrothermal–enzymatic (cellulase–pectinase–protease)–ultrasonication; HU, mild hydrothermal–ultrasonication without enzymes.

SEM micrographs of HEU‐C ULP (Figure 2) revealed an irregular morphology characterized by rough, porous surfaces and fragmented, sheet‐like structures. This morphology is likely influenced by the thermal treatment applied during extraction. Heating at 70°C–80°C was applied to weaken and partially denature cell wall structures, thereby facilitating ulvan release from the tissue matrix. However, thermal exposure may also induce partial depolymerization, resulting in shorter polysaccharide chains. This interpretation is supported by the overall morphology observed across samples, where particles appear small, irregular, and more fragmented. Similar features have been reported previously, where semipurified ulvan exhibited an amorphous, nonsmooth texture in SEM images [58].

In addition to thermal effects, ultrasonic treatment (20–40 kHz) likely contributed to morphological disruption through acoustic cavitation. The collapse of microbubbles generates localized high shear forces capable of breaking cell walls and fragmenting polysaccharides into smaller particles. These cavitation effects may also produce visible surface pores, which are consistent with the microstructural features observed.

The enzymatic hydrolysis step using cellulase may not directly cleave ulvan chains; however, it degrades cellulose and hemicellulose components of the *Ulva* cell wall, thereby enhancing ulvan liberation from the matrix. Consequently, particles produced under HEU‐C conditions appeared less aggregated and exhibited a relatively smoother yet still amorphous and noncrystalline morphology. When cellulase was combined with pectinase and protease in other treatments, enzymatic synergy likely intensified cell wall disruption and reduced particle aggregation, resulting in a more heterogeneous and amorphous structure. These structural alterations are consistent with previous reports describing ulvan obtained through combined physical–enzymatic extraction processes [27]. In contrast, HU samples exhibited a denser and more compact morphology with fewer visible pores, indicating limited structural disruption in the absence of enzymatic assistance.

Overall, SEM observations confirm that the combined thermal, ultrasonic, and enzymatic treatments induced substantial morphological modifications, yielding amorphous, porous, and finely fragmented ulvan particles. Such structural changes may enhance solubility and increase the accessibility of functional groups (–SO_3_^−^ and –COOH), which are critical for ulvan bioactivity.

### 3.5. FTIR Analysis

**Figure 3:**
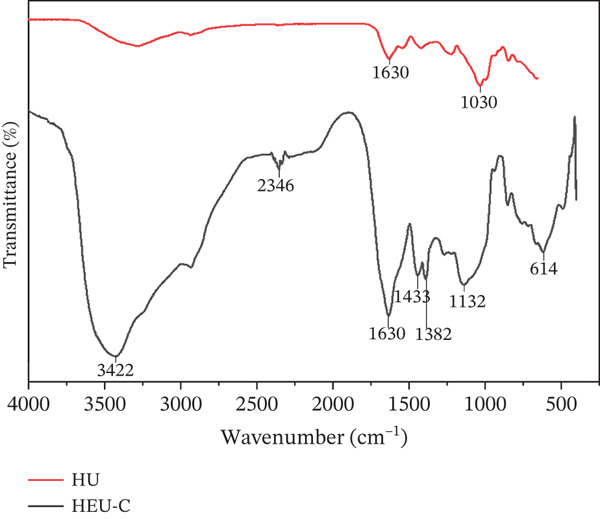
Fourier‐transform infrared (FTIR) spectra of ulvan extracted using HEU‐C (cellulase‐assisted hydrothermal–ultrasonication) and HU (nonenzymatic hydrothermal–ultrasonication). Key absorption bands corresponding to hydroxyl (O–H), carboxyl (C=O), glycosidic (C–O–C), and sulfate (S=O) functional groups are indicated, illustrating differences in chemical structure and functional group integrity between the enzymatic and nonenzymatic extracts.

Given that HEU‐C ULP demonstrated the highest chemical composition and antioxidant activity, FTIR spectroscopy was performed to compare HEU‐C ULP with the nonenzymatic HU ULP, identifying differences in structural features and functional group integrity (Figure 3). The FTIR spectra of HEU‐C ULP displayed characteristic ulvan‐associated bands, including sulfate ester groups (S=O), hydroxyls (O–H), carboxylates (C=O), and glycosidic linkages (C–O–C), consistent with previous reports [59, 60]. In contrast, the nonenzymatic HU ULP exhibited higher absorption intensities across most functional groups, reflecting a relatively more intact native ulvan structure. Prominent peaks observed at 840–850 cm^−1^ (*β*‐anomeric C–H bending), 1028–1217 cm^−1^ (C–O–C and S=O stretching), and 1413–1538 cm^−1^ (CH_2_ and COO^−^ bending) further confirm the preservation of glycosidic and sulfate signatures in HU ULP. Conversely, the HEU‐C ULP displayed attenuated peak intensities, particularly at 3422 cm^−1^ (O–H) and 1628 cm^−1^ (C=O), indicating partial structural disruption. These reductions likely arise from selective enzymatic hydrolysis, the weakening of hydrogen‐bonding networks, and oxidative modifications occurring during the intensified extraction process.

The sulfate groups (S=O), detected at 1134 and 1217 cm^−1^, are critical contributors to ulvan′s antioxidant activity through electron‐donating behavior in redox reactions. Consequently, a decrease in peak intensity at these wavelengths may indicate a reduction in localized sulfate density or changes in sulfate accessibility. However, the HEU‐C extract exhibited higher antioxidant activity in the present study, suggesting that factors beyond sulfate abundance, such as uronic acid content, molecular conformation, and functional group accessibility, also contribute to radical scavenging performance. Characteristic sulfate absorption bands in ulvan typically appear between 1250 and 1230 cm^−1^, with additional ester sulfate signals at 850–860 and 790–800 cm^−1^ [36]. Table 2 summarizes the observed peaks and their corresponding structural assignments. The intensity of these bands is strongly influenced by the abundance and spatial distribution of sulfate moieties within the polymer matrix.

**Table 2:** Comparison of FTIR absorption bands and functional group interactions between ULP with treated HU and HEU‐C.

Theoretical wavenumber (cm^−1^)	Theoretical functional group	Sample (cm^−1^)		Interpretation
		HU	HEU‐C	
~3400	O–H stretching	3272.25	3422.92	Indicates the presence of hydroxyl groups of monosaccharides
~2930	C–H stretching	2931.49	2351.05	Shows the aliphatic chain of sugar residues
~1650	C=O stretching (carboxylate)	1413.40, 1538.13, 1627.71	1628.34	Indicates uronic acid in the ulvan structure
~1250–1270	S=O asymmetric stretching	1217.39	1384.93 1433.46	Sulfate group, the main marker of sulfated polysaccharides
~1050–1100	C–O–C stretching	1028.32	1134.14	Glycosidic bonds between monosaccharides
~890	C–H bending (*β*‐anomeric)	840.94	846.81 614.47	Shows the *β* configuration of the glycosidic bond

Abbreviations: HEU‐C, mild hydrothermal–enzymatic (cellulase)–ultrasonication; HU, mild hydrothermal–ultrasonication without enzymes.

Previous studies have reported that ultrasonication‐assisted extraction can enhance S=O peak intensities compared to conventional methods by increasing the exposure of these groups [61]. For EAE, the S=O intensity may decrease due to partial desulfation or the loss of highly sulphated low‐molecular‐weight fragments during precipitation. However, enzymes with selective cleavage activity may also expose sulfate sites previously embedded within the polysaccharide network, occasionally leading to enhanced spectral intensities. Shifts in peak positions may also occur as a consequence of changes in the chemical environment, such as altered hydrogen bonding or conformational rearrangements of the ulvan backbone.

Carboxyl groups from uronic acids (e.g., glucuronic and iduronic acid) also contribute to antioxidant capacity via proton donation and the chelation of transition metal ions, thereby inhibiting hydroxyl radical formation [60, 62]. The presence of these uronic acids further improves solubility and chain flexibility, enhancing interaction with free radicals. Collectively, these results suggest that while enzymatic treatment induces minor structural modifications, it optimizes the release of functional uronate‐rich fractions. To complement these findings, further structural elucidation using nuclear magnetic resonance spectroscopy (1H and 13C NMR) and gel permeation chromatography (GPC) would be valuable to determine precise anomeric configurations and molecular weight distributions, respectively.

### 3.6. Effects of ULP Supplementation at Different Treatments and Concentrations on the Viability of Probiotic Candidates

The prebiotic effects of ULP were evaluated at different concentrations (1%, 2.5%, and 5%) and hydrolysis treatments (HEU‐C and HEU‐Mix) using probiotic candidates cultivated in glucose‐free modified MRSB. In general, both HEU‐C‐ and HEU‐Mix‐derived ULP hydrolysates enhanced bacterial viability after 24 h, although the response was strongly strain‐dependent and varied across substrate concentrations (Table 3).

**Table 3:** Viability of probiotic candidates at 0 and 24 h in media supplemented with ULP (1%–5%) under HEU‐C and HEU‐Mix treatments.

Probiotic candidate	Prebiotic concentration	Viability at 0 h (log CFU/mL)	Viability at 24 h (log CFU/mL, *m* *e* *a* *n* ± *S* *D*)		*Δ*Viability (log CFU/mL)	
			HEU‐C	HEU‐Mix	HEU‐C	HEU‐Mix
RAL43	1% ULP	7.12	8.47 ± 0.02^bcA^	8.47 ± 0.02^dA^	1.35	1.35
	2.5% ULP	7.19	8.82 ± 0.14^cB^	8.09 ± 0.05^bB^^∗^	1.64	0.91
	5% ULP	7.92	8.73 ± 0.35^bcB^	8.56 ± 0.06^dA^	0.80	0.64
	FOS	6.89	8.28 ± 0.06^bAB^	8.28 ± 0.06^cAB^	1.38	1.39
	NC	6.05	6.28 ± 0.08^aA^		0.23	
RAL25	1% ULP	7.26	8.30 ± 0.52^bA^	8.30 ± 0.52^bA^	1.03	1.25
	2.5% ULP	7.21	8.49 ± 0.01^bA^	8.37 ± 0.38^bB^	1.28	1.45
	5% ULP	8.98	8.05 ± 0.05^bA^	8.05 ± 0.05^bA^	‐0.93	‐0.93
	FOS	6.91	8.42 ± 0.04^bB^	8.42 ± 0.04^bB^	1.52	1.52
	NC	6.21	6.44 ± 0.11^aAB^		0.24	
SWR14	1% ULP	6.99	8.48 ± 0.06^cA^	8.31 ± 0.35^bA^	1.49	1.43
	2.5% ULP	6.93	8.80 ± 0.05^dB^	8.64 ± 0.27^bB^	1.87	1.94
	5% ULP	7.34	8.21 ± 0.18^bAB^	8.25 ± 0.13^bA^	0.87	0.96
	FOS	6.56	8.42 ± 0.06^bcB^	8.42 ± 0.06^bB^	1.86	1.87
	NC	6.36	6.52 ± 0.01^aB^		0.15	
MIX	1% ULP	8.04	8.47 ± 0.02^bcA^	7.93 ± 0.85^abA^	0.43	0.23
	2.5% ULP	8.11	8.40 ± 0.17^bcA^	6.74 ± 0.38^abA^^∗^	0.28	‐1.09
	5% ULP	7.70	8.57 ± 0.11^cAB^	7.97 ± 0.89^abA^	0.87	0.62
	FOS	6.30	8.21 ± 0.10^bA^	8.21 ± 0.10^bA^	1.91	1.92
	NC	6.30	6.51 ± 0.02^aB^		0.21	

*Note:* Different lowercase superscript letters (a–c) indicate significant differences in probiotic viability among PUL concentrations within each strain (*p* < 0.05). Different uppercase superscript letters (A–C) indicate significant differences among strains at the same PUL concentration (*p* < 0.05).

Abbreviations: HEU‐C, hydrothermal–enzymatic (cellulase)–ultrasonication; HEU‐Mix, hydrothermal–enzymatic (cellulase–pectinase–protease)–ultrasonication; NC = no carbon source.

*Significant differences between HEU‐C and HEU‐Mix treatments based on independent‐samples *t*‐tests (*p* < 0.05).

Across all strains tested, supplementation with 2.5% ULP resulted in the greatest enhancement of bacterial viability. Among the single strains, RAL43 exhibited the most pronounced response under the HEU‐C condition, with a viability increase of 1.64 log CFU/mL, exceeding the performance of the FOS control. In contrast, under HEU‐Mix treatment, the stimulatory effect on RAL43 was markedly reduced (*Δ*0.91 log CFU/mL). This differential response suggests that cellulase‐mediated modification of the ulvan matrix improves carbohydrate accessibility for RAL43, whereas more extensive depolymerization under mixed‐enzyme hydrolysis (cellulase–pectinase–protease) may disrupt substrate architecture or generate competing nutrient fractions that diminish its prebiotic efficacy.

RAL25 and SWR14 exhibited more consistent responses, showing comparable viability increases under both HEU‐C and HEU‐Mix treatments (*Δ*1.28–1.45 and *Δ*1.87–1.94 log CFU/mL, respectively). Their growth approached levels observed with FOS and did not display strong matrix‐dependent variation, indicating broader metabolic compatibility with ULP‐derived carbohydrates regardless of the hydrolysis mode. However, increasing the ULP concentration to 5% led to reduced viability across all strains, with the most pronounced decline observed for RAL25 (*Δ*–0.93 log CFU/mL). This suppression at higher concentrations suggests that excessive polysaccharide availability may impose physiological stress, potentially due to increased medium viscosity, osmotic pressure, or accumulation of sulfated oligosaccharides that interfere with metabolic homeostasis.

The observed concentration‐dependent effects are consistent with previous reports on ulvan‐derived substrates. Krangkratok et al. [23] reported that ulvan oligosaccharides at 1% significantly enhanced the growth of *Lb. acidophilus* and *Lb. plantarum*, whereas higher concentrations did not further improve viability. Similarly, Jagtap et al. [63] demonstrated that ulvan hydrolysates improved *Lb. acidophilus* growth beyond that achieved with FOS but only within an optimal substrate concentration range. Collectively, these findings reinforce the notion that substrate accessibility and metabolic compatibility, rather than substrate abundance alone, govern the prebiotic efficacy of ulvan‐based carbohydrates.

Strain‐specific differences in response to ULP supplementation likely reflect underlying genetic and enzymatic capacities. Seong et al. [64] reported that only certain LAB, such as *Lb. plantarum* and *Lb. rhamnosus*, possess key enzymes, including *α*‐L‐rhamnosidase and *β*‐glucuronidase, enabling the utilization of ulvan‐derived sugars like sulfated rhamnose, glucuronic acid, and iduronic acid. The variability observed among the isolates in this study is therefore attributable to differences in metabolic pathways and enzymatic repertoires. Further genomic or transcriptomic profiling would be valuable to elucidate the specific mechanisms underlying ulvan utilization by these strains.

In contrast to the single‐strain cultures, the mixed‐culture consortium (RAL43+RAL25+SWR14) exhibited reduced total viability, particularly at 2.5% ULP under the HEU‐Mix treatment (*Δ*–1.09 log CFU/mL). This decline likely reflects increased substrate competition, metabolic interference, or environmental stress under coculture conditions rather than strain‐specific dominance. Consistent with this observation, Seong et al. [64] reported that ulvan selectively promotes specific microbial taxa, while nonadapted species may show limited responses. Within the constraints of total plate count enumeration, the present findings suggest that ULP exerts selective effects at the community level rather than uniformly enhancing mixed‐culture viability. While ULP effectively supports the viability of individual strains, particularly RAL43 under optimized hydrolysis, its ability to sustain multistrain consortia appeared limited under the conditions tested. Accordingly, based on its robust and consistent performance in single‐strain fermentations, RAL43 emerges as the most promising candidate for future synbiotic applications involving ULP.

Importantly, the relatively high initial inoculation level used in this study (~7.0–8.5 log CFU/mL at 0 h) indicates that the in vitro fermentation assay primarily reflects bacterial survival and metabolic activity rather than exponential growth. This experimental design ensured sufficient metabolic activity for detecting carbohydrate utilization and SCFA production within a 24‐h period. Under these conditions, changes in viability are more appropriately interpreted as indicators of strain‐specific substrate adaptation, metabolic efficiency, and competitive interactions, particularly in mixed cultures, rather than classical growth‐promoting effects. Accordingly, the prebiotic effects of ULP described in this study should be understood in terms of fermentative performance and metabolic resilience in response to ulvan supplementation.

### 3.7. SCFA Production During Fermentation of HEU‐C ULP by *Lb. rhamnosus* RAL43

**Figure 4:**
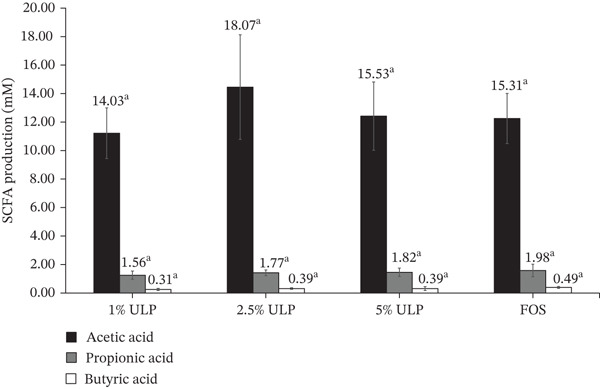
Short‐chain fatty acid (SCFA) concentrations produced by *L. rhamnosus* RAL43 after 24‐h fermentation with HEU‐C *Ulva lactuca* polysaccharides (ULPs) at different concentrations, compared with FOS as control. Values are mean ± SD (*n* = *X*). Different superscript letters indicate significant differences between concentrations (*p* < 0.05).

Fermentation of HEU‐C ULP by *Lb. rhamnosus* RAL43 yielded a consistent SCFA profile dominated by acetic acid, with substantially lower concentrations of propionic and butyric acids (Figure 4). Although minor numerical differences were observed among treatments, no statistically significant variation was detected across ULP concentrations for any SCFA measured (*p* > 0.05). Acetic acid concentrations ranged from 14.03 to 18.07 mM, whereas propionic and butyric acids remained comparatively low at 1.56–1.98 and 0.31–0.49 mM, respectively. The similarity in SCFA profiles between ULP treatments and the FOS control indicates that HEU‐C ULP supports fermentative metabolic activity comparable to that of a well‐established prebiotic substrate.

The predominance of acetate is consistent with the established metabolic characteristics of LAB, in which acetate typically represents the major fermentation end‐product, while propionate and butyrate production are strain‐dependent and generally occur at lower levels [38, 65]. Chang et al. [65] similarly reported that yogurt formulations containing *Bifidobacterium bifidum*, *Lb. acidophilus*, or *Lb. gasseri* produced substantially higher acetate concentrations than propionate or butyrate, with the latter often remaining unchanged across different inoculation schemes. In LAB, acetate formation is primarily associated with the phosphoketolase pathway [66], whereas propionate‐ and butyrate‐producing pathways are less clearly defined and are considered metabolically constrained [65]. Consistent with this, *Lb. rhamnosus* is not generally regarded as a classical butyrate‐producing bacterium.

In the present study, butyrate identification was based on retention time comparison with authenticated standards. However, no additional confirmatory analyses (e.g., coinjection, LC–MS validation, or spike recovery assay) were performed. Therefore, the low concentrations detected may partly reflect coeluting compounds or background signal originating from the fermentation medium. Despite their low abundance, propionate and butyrate remain functionally relevant due to their reported roles in gut barrier protection, immune modulation, lipid metabolism, and suppression of opportunistic pathogens [18, 20, 28, 67]. Collectively, these observations support growing evidence that ulvan‐based substrates can be selectively metabolized by LAB, extending their fermentative capacity beyond conventional dairy‐derived carbohydrates.

When compared with previous studies, the acetate concentrations observed in this work were lower than those reported for *Lb. rhamnosus* R23 fermenting conventional prebiotic substrates such as glucose (41.42 mM), galactooligosaccharides (38.56 mM), or inulin hydrolysate (38.22 mM) [68]. These differences likely reflect variations in substrate complexity, monosaccharide accessibility, and degree of polymerization. While glucose and commercial oligosaccharides provide readily assimilable carbohydrates, ulvan consists primarily of sulfated rhamnans and uronic acids that may require specific depolymerization prior to fermentation, resulting in slower but more sustained SCFA production. This suggests that carbohydrate accessibility, rather than substrate abundance alone, may represent the primary limiting factor in SCFA conversion efficiency. Notably, the comparable SCFA output between ULP and FOS highlights the potential of marine‐derived polysaccharides as alternative carbon sources for LAB fermentation.

Although no dose‐dependent shift in SCFA production was observed, the consistent SCFA output across ULP concentrations indicates that *Lb. rhamnosus* RAL43 can metabolize HEU‐C ULP without inhibitory effects. Future optimization strategies, such as controlled nitrogen supplementation, metabolic pathway engineering, or coculturing with butyrate‐producing taxa, may further enhance metabolic diversity and increase total SCFA yield. Overall, these results demonstrate that HEU‐C ULP is a fermentable substrate capable of supporting stable SCFA production by *Lb. rhamnosus* RAL43, underscoring its potential application as a novel prebiotic ingredient for microbiome‐targeted functional food development.

### 3.8. Soluble Sugar Changes During Fermentation of HEU‐C ULP by *Lb. rhamnosus* RAL43

**Figure 5:**
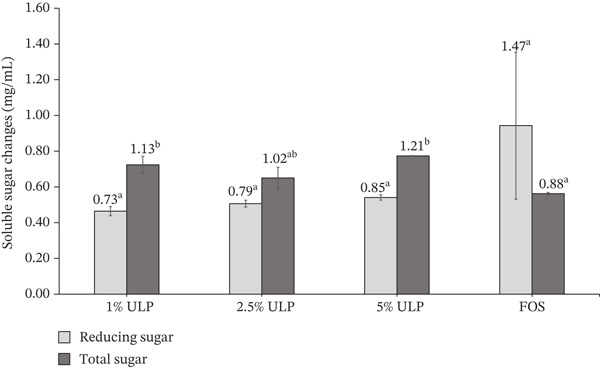
Remaining reducing sugar and total soluble sugar concentrations after 24‐h fermentation of HEU‐C *Ulva lactuca* polysaccharides (ULPs) by *L. rhamnosus* RAL43 at different concentrations, compared with FOS. Data are expressed as mean ± SD (*n* = *X*). Bars with different letters differ significantly (*p* < 0.05).

After 24 h of incubation with *Lb. rhamnosus* RAL43, measurable differences in residual soluble sugar fractions were observed across treatments (Figure 5). Residual reducing sugar concentrations in ULP‐supplemented media ranged from 0.73 to 0.85 mg/mL, whereas the FOS control retained a higher level (1.47 mg/mL), although this difference was not statistically significant. In contrast, total soluble sugar levels exhibited a distinct pattern, with ULP treatments retaining significantly higher concentrations (1.02–1.21 mg/mL) compared to the FOS control (0.88 mg/mL).

The higher residual reducing sugar detected in the FOS treatment likely reflects active oligosaccharide hydrolysis rather than incomplete substrate utilization. Reducing sugar assays quantify free reducing ends; thus, the enzymatic cleavage of FOS by *β*‐fructofuranosidase releases fructose monomers that contribute to the measured reducing sugar levels. Consequently, elevated residual reducing sugar does not necessarily indicate reduced metabolic activity but may instead represent the transient accumulation of hydrolysis products prior to cellular uptake. In contrast, ULP‐derived carbohydrates likely undergo slower or partial depolymerization, potentially generating lower levels of freely available reducing sugars during fermentation. Accordingly, the lower residual reducing sugar observed in ULP treatments may reflect limited monosaccharide release rather than more extensive carbohydrate depletion. Importantly, the comparable SCFA production observed in both ULP and FOS treatments confirms active fermentation despite variations in residual sugar levels, indicating that hydrolysis and metabolic assimilation proceed at different rates. These findings highlight that reducing sugar quantification alone does not fully capture fermentation efficiency, particularly for complex oligosaccharide and polysaccharide substrates.

Increasing the ULP concentration did not result in a proportional reduction in residual soluble sugars, suggesting that fermentation dynamics differ from the rapid utilization typically observed for readily fermentable substrates like FOS. The relatively modest decrease in reducing sugars, despite detectable metabolic activity, indicates that *Lb. rhamnosus* RAL43 may not rely exclusively on freely available simple sugars. Instead, carbohydrate metabolism likely involves the gradual utilization of partially hydrolyzed or structurally complex ulvan fractions. This interpretation is consistent with the metabolic flexibility described for LAB, whereby substrate complexity, enzyme accessibility, and carbon source availability influence utilization patterns [69].

Taken together, the limited availability of fermentable monosaccharides in ULP‐supplemented media, alongside sustained microbial metabolic activity, suggests that HEU‐C‐derived ulvan functions as a slowly fermentable substrate. This is further supported by the consistent SCFA production observed across all ULP concentrations, indicating ongoing fermentation even when rapidly utilizable sugar pools were constrained. Similar observations have been reported by Seong et al. [64], who noted that sulfated and structurally complex seaweed polysaccharides are generally fermented more slowly than conventional prebiotics such as FOS.

Nevertheless, as the monosaccharide and oligosaccharide compositions of the fermented ULP fractions were not characterized in this study, it remains unclear whether the observed changes primarily reflect direct utilization of existing soluble sugars, partial enzymatic depolymerization of ulvan, or a combination of both mechanisms. Further structural characterization, such as chromatographic profiling of carbohydrate fractions before and after fermentation, is required to elucidate the dominant metabolic pathways and confirm the contribution of ulvan degradation to fermentative activity.

## 4. Conclusion

This study demonstrates that the extraction strategy plays a decisive role in determining the yield, chemical composition, functional properties, and prebiotic potential of ULPs. Among the evaluated approaches, the mild hydrothermal–enzymatic–ultrasonication process using cellulase (HEU‐C) yielded the most favorable overall profile. This was evidenced by a significantly higher extraction yield, increased levels of total polysaccharides, reducing sugars, and uronic acids, as well as superior antioxidant activity. While the multienzyme system (HEU‐Mix) induced structural modifications, no clear synergistic advantage over the single‐enzyme strategy was observed. In contrast, the nonenzymatic hydrothermal–ultrasonication method (HU) resulted in the lowest yield and functional performance, despite maintaining a relatively higher sulfate content.

In vitro fermentation assays further confirmed that ULP effectively supports the viability of probiotic candidates, including *Lb. rhamnosus*, *Lb. plantarum*, and *Lc. lactis*. Growth responses were comparable to or exceeded those observed with FOSs, depending on strain specificity and supplementation levels. Furthermore, ULP supplementation sustained SCFA production, predominantly acetic acid, indicating active microbial metabolism and reinforcing the functional relevance of ULP as a fermentable substrate.

Collectively, these findings highlight *U. lactuca* from Indonesian coastal waters as a promising yet underutilized source of bioactive marine polysaccharides with prebiotic potential. Future studies should focus on molecular‐level structural elucidation, optimization of scalable extraction processes, and in vivo validation of gut microbiota modulation. In parallel, the incorporation of ULP into synbiotic, nutraceutical, and functional food formulations remains essential to translate these findings into practical applications for human health.

## Author Contributions

Yuliana Tandi Rubak: conceptualization, methodology, formal analysis, data curation, writing (original draft), and project administration. Herianus J. D. Lalel: conceptualization, supervision, and review and editing. Budiana I. Gusti M. Ngurah: conceptualization, supervision, and review and editing. Welem Linggi Turupadang: conceptualization, methodology, formal analysis, data curation, and writing—original draft. Fenny Amilia Mahara: conceptualization, methodology, formal analysis, data curation, and writing—original draft. Dandy Yusuf: conceptualization, methodology, formal analysis, data curation, and writing—original draft.

## Funding

This study was funded by the Ministry of Higher Education, Science and Technology, Republic of Indonesia, 092/C3/DT.05.00/PL/2025.

## Conflicts of Interest

The authors declare no conflicts of interest.

## Data Availability

All data supporting the findings of this study are included within the manuscript.
